# Supporting Preschoolers’ Mental Health and Academic Learning through the PROMEHS Program: A Training Study

**DOI:** 10.3390/children10061070

**Published:** 2023-06-16

**Authors:** Elisabetta Conte, Valeria Cavioni, Veronica Ornaghi, Alessia Agliati, Sabina Gandellini, Margarida Frade Santos, Anabela Caetano Santos, Celeste Simões, Ilaria Grazzani

**Affiliations:** 1 “R. Massa” Department of Human Sciences for Education, University of Milano-Bicocca, 20126 Milan, Italy; valeria.cavioni@unifg.it (V.C.); veronica.ornaghi1@unimib.it (V.O.); alessia.agliati@unimib.it (A.A.); sabina.gandellini@unimib.it (S.G.); ilaria.grazzani@unimib.it (I.G.); 2 Department of Humanities, Literature, Cultural Heritage, Education Sciences, University of Foggia, 71121 Foggia, Italy; 3 Faculty of Psychology, University of Milano-Bicocca, 20126 Milan, Italy; 4 Department of Education, Social Sciences and Humanities, Faculty of Human Kinetics, University of Lisbon, 1495-751 Lisbon, Portugal; m_margarida_santos@hotmail.com (M.F.S.); anabela.caetano.s@gmail.com (A.C.S.); csimoes@fmh.ulisboa.pt (C.S.); 5 Environmental Health Institute (ISAMB), Faculty of Medicine, University of Lisbon, 1649-028 Lisbon, Portugal

**Keywords:** social-emotional learning, prosocial behavior, problem behaviors, academic outcomes, school mental health, PROMEHS

## Abstract

There is compelling evidence that early school intervention programs enhance children’s development of life skills, with a positive knock-on effect on their behaviors and academic outcomes. To date, most universal interventions have displayed gains in children’s social-emotional competencies with a limited reduction in problem behaviors. This may depend on programs’ curricula focused to a greater extent on preschoolers’ social-emotional competencies rather than problem behaviors. Promoting Mental Health at Schools (PROMEHS) is a European, school-based, universal mental health program explicitly focused on both promoting students’ mental health and preventing negative conduct by adopting a whole-school approach. In this study, we set out to evaluate the effectiveness of the program for Italian and Portuguese preschoolers. We recruited 784 children (age range = 4–5 years), assigning them to either an experimental group (six months’ participation in the PROMEHS program under the guidance of their teachers, who had received ad hoc training) or a waiting list group (no intervention). We found that PROMEHS improved preschoolers’ social-emotional learning (SEL) competencies, prosocial behavior, and academic outcomes. The more practical activities were carried out at school, the more children’s SEL competencies increased, and the more their internalizing and externalizing behaviors decreased. Furthermore, marginalized and disadvantaged children were those who benefited most from the program, displaying both greater improvements in SEL and more marked decreases in internalizing problems compared to the rest of the sample.

## 1. Introduction

Crucially, the first six years of life lay the ground for children’s cognitive, physical, linguistic, and social-emotional development. Early childhood experiences in the home and in extra-familial settings can shape the foundations of subsequent developmental stages [1]. Given that both innate and environmental factors play a role in development, adults can foster abilities and competencies with the potential to influence children’s mental health and habits over time. While parents bear much of the responsibility for this process and can impact their child’s adjustment [2,3], it must be recognized that teachers and educators can also influence children’s overall development. Currently, almost a third of European children under 3 years of age are enrolled in formal childcare [4] while 93% of children over the age of 3 attend early childhood education and care services [5]. Thus, for the majority of children, preschool is the first educational service to be accessed outside the home, making educational facilities ideal venues for supporting children’s acquisition of life skills.

Among such skills, social-emotional competencies have been shown to protect against mental health issues [6,7,8,9], highlighting the importance of early social-emotional learning (SEL). SEL is defined as the process through which individuals acquire the knowledge, skills, and attitudes implicated in five inter-related social-emotional competencies: self-awareness, self-management, social awareness, relationship skills, and responsible decision-making [10,11]. These five social-emotional competencies retain their core status across the life span, but their content changes as a function of age-related developmental tasks [12].

Self-awareness includes the ability to accurately recognize one’s own internal states, strengths, and weaknesses and how these can influence one’s behaviors. After the age of 1 year, children begin—amongst other self-aware behaviors—to name themselves (using the proper noun “I” or the terms “mine”, “me”, “myself”), recognize themselves (e.g., in the mirror or in photographs), and express their internal states [13,14]. During the preschool years, they display additional signs of self-awareness, including increasing ability to label their more complex feelings, to identify both internal and external causes for their emotions, to describe their interests and what they are good and not good at, and to reflect on the reasons why they act in a certain way, all thanks to the ongoing parallel development of their linguistic and social cognition skills [15,16,17].

Another competence that largely concerns the individual is self-management, which encompasses the ability to efficiently manage one’s own emotions, thoughts, and behaviors, as well as to set and act to achieve goals. Mastering these abilities makes it possible to control automatic and inappropriate responses, which are common during the first years of life. Children early acquire self-regulation strategies (e.g., thumb sucking, hiding, turning away in situations where they do not feel at ease), but in most cases they need help from adults to compensate for their as-yet basic abilities [18]. During the preschool years, they gradually acquire increasingly complex strategies (such as explaining their own desires, goals, or needs, engaging with others’ points of view and feelings during conflicts, being persistent in completing difficult tasks, distraction) for managing stressful situations by themselves and controlling their reactions when interacting with peers. Initially, children may struggle to implement these strategies successfully and thus continue to require co-regulation from their caregivers in order to calm down, achieve pre-defined goals, or adapt their behaviors [19,20].

Another social-emotional competence is responsible decision-making, which includes the ability to realistically evaluate the consequences of one’s choices for self and others. It implies the application of moral and ethical principles when deciding how to act in everyday life situations. For example, a preschooler might judge that stealing a toy from another child would be unfair and could lead to a fight, and thus choose to seek out another toy or object; or, they could reprimand a peer at the playground for climbing backwards up the slide rather than taking the ladder, pointing out that this is dangerous, or unfair to the other children queuing to use the slide. These examples reflect preschoolers’ understanding of social norms, values, shared commitments within a community, and reciprocal respect, which helps them to successfully adjust to their social environment. In the early years of life, children internalize the rules of adults about what is right and wrong and act accordingly, but from 3 years onwards they demonstrate a complex understanding of their own and actively enforce social norms. For example, they correct one another’s behavior, both during games with explicit rules and during pretend play [21,22]. Furthermore, they protest when a third party’s property rights are violated [23]. Thus, children enforce social norms whether or not they are directly involved in an interaction, implying an early tendency to act appropriately in social situations with a view to maintaining the integrity and wellbeing of their social group [24].

To morally judge another individual and evaluate the consequences of that person’s actions, the child needs to acquire and integrate information about what other people think, desire, want to achieve, etc. [25]. Understanding the minds of others is a prerequisite for social awareness, which is defined as the ability to adopt the perspectives of others and be empathetic toward others, including those with different personal and socio-cultural backgrounds. These capacities develop very early [26], and by the preschool years, the child has acquired the ability to explicitly reflect on the actions of others and to explain the behavior of interlocutors by inferring their feelings, thoughts, beliefs, perceptions, etc. [27].

By adopting others’ perspectives and experiencing others’ mental states, children further enhance their relationship skills, which encompass the tendency to create, maintain, and repair positive relationships. Indeed, they gradually acquire the ability to effectively communicate, listen to others, solve conflicts, cooperate with others, and offer and request help as appropriate. Having a wide variety of interactions both at home and in extra-familial contexts helps children to experience different kinds of relationships, both with peers and adults, and to identify effective strategies. For example, the child will learn to negotiate, identify a compromise, or apologize in order to solve conflicts with siblings or peers. They will learn to offer help or comfort to someone who is in distress (e.g., by drawing, hugging, or listening to an interlocutor). Thanks to attitudes and behaviors such as these, children are perceived as friendly, and are more likely to be popular with their peers and less likely to suffer peer rejection [28].

Although the five social-emotional competencies just outlined are acquired spontaneously, they are also malleable and susceptible to improvement by means of early intervention [29,30,31]. Children generally spend a significant amount of time at school, while preschool educational settings allow teachers more freedom to integrate SEL practices and activities into their daily routine. There is evidence that early interventions are more effective in the promotion of social-emotional competencies compared to those carried out with older students [32]. Furthermore, as illustrated in the next section, participation in SEL programs produces long-lasting benefits, including the prevention of negative psychological and behavioral outcomes [33].

### 1.1. SEL Competencies and Mental Health

There is evidence that children’s mental health—a multidimensional construct that encompasses multiple aspects of psychological and social functioning [34,35]—is associated with their development of social-emotional competencies. Indeed, more advanced social-emotional competencies are associated with enhanced positive outcomes (e.g., prosocial behaviors) and also help to prevent problematic behaviors such as internalizing (e.g., anxiety, social withdrawal) and externalizing (e.g., aggression, hyperactivity) issues. For example, numerous studies have identified positive associations between children’s social awareness and their propensity to engage in prosocial conducts such as sharing, helping, comforting, mediating, and cooperating [36,37,38,39]. Self-management also has been associated with positive behavioral outcomes [40,41]. Gender-related differences have been reported, with girls obtaining higher ratings for both SEL competencies and prosocial behaviors compared to boys [38,40]. With regard to problem behaviors in preschoolers, children’s SEL skills have been reported to be negatively associated with both internalizing and externalizing problems [42,43,44]. In this relationship, gender may play a role. Maguire et al. identified similar overall patterns for boys and girls (aged 4–6 years) in terms of the association between emotional competencies and problem behaviors, but poorer emotion understanding was associated with more externalizing behaviors in boys and more internalizing behaviors in girls. Furthermore, males engaged in fewer prosocial and more externalizing behaviors than did females [45].

Further evidence for the association between social-emotional competencies and mental health has been outlined in studies on the impact of SEL programs and other forms of intervention focused on emotion [31,46,47]. Meta-analyses and systematic reviews have usefully collated and reinforced the conclusions of primary studies, showing that SEL programs significantly increased positive social behaviors and reduced both internalizing and externalizing problems [8,48,49,50], with greater effects in younger students [32,51], thus adding to the evidence that social-emotional competencies protect against the onset of mental health issues.

### 1.2. Children’s Mental Health and Early Learning Outcomes

An extensive body of research suggests that preschoolers’ mental health is linked with several early learning outcomes, both concurrently and predictively over time [52]. Children who start kindergarten with greater competence in managing their own and others’ emotions, and the ability to establish and maintain healthy relationships with peers and adults, display successful early school adjustment [53,54,55,56]. Specifically, preschoolers’ social-emotional competencies are strongly associated with their level of school readiness (e.g., literacy and numeracy skills), even after controlling for cognitive ability and family background [57,58,59]. Conversely, preschoolers who lack developmentally appropriate social-emotional competencies tend to participate less in classroom activities, displaying poorer motivation and greater difficulty performing early academic tasks [60,61]. Notably, boys tend to score more poorly on assessments of these behaviors and attitudes at school [62]. Deficits may persist across the elementary and secondary years, increasing the risk of subsequent school dropout [63,64,65]. In general, studies such as those outlined have suggested that early intervention in support of children’s mental health is valuable because it not only enhances students’ wellbeing but also boosts their levels of achievement in the short and long term [66,67].

### 1.3. School-Based Interventions in Preschool Settings and the PROMEHS Program

Over the last three decades, an increasing number of evidence-based mental health programs for students—a type of intervention that was initially developed in the United States—have been conducted worldwide [34]. Mental health programs can be designed to promote different social-emotional abilities (e.g., communication, empathy) and prevent specific behaviors, difficulties, or disorders (e.g., bullying, anxiety, violence), depending on their theoretical underpinnings and specific goals, and on whether they are delivered as universal (i.e., Tier 1) or targeted (i.e., Tier 2) interventions—that is to say, whether they are for all students or only for those displaying signs of mental health difficulties [68,69,70].

Most mental health interventions for preschoolers have focused on fostering social-emotional competencies, offering children psychological, social, cultural, and physical resources for coping with stress and challenges and for building psychological wellbeing [71,72]. As such, these programs have typically been developed to enhance protective factors for mental health rather than to reduce or address existing challenging behaviors [49]. A large meta-analysis by Blewitt et al. of 63 universal interventions carried out in early childhood education and care centers showed that participation was associated with major improvements in positive proximal outcomes (e.g., social-emotional competencies), whereas smaller or no effects were observed in terms of decreased distal outcomes (e.g., challenging behaviors) [73]. The learning process necessary to display positive behaviors may require more time because the child has to integrate the new skills and adjust behaviors into everyday life. Thus, it is possible that distal outcomes may be delayed and could be more strongly appreciated after a few months, especially if parents are involved in the implementation and should change their attitudes and behaviors as well [74].

In this regard, multi-focused interventions that combine initiatives for children, teachers, and parents have showed to be effective in promoting children’s competencies and decreasing their problem behaviors [75,76]. Adults’ joint work and alignment on the best practices can enhance children’s development and the generalization of acquired skills to their everyday lives. In this perspective, principals also play a crucial role in the school setting because they can provide foundational support to the school community and reinforce the best practices. Therefore, the involvement of the whole school community should be valued in school-based interventions [77].

Additionally, as universal interventions address all students, at-risk children can benefit from the programs that are delivered at school. Most previous universal interventions on preschoolers have also evaluated the effectiveness of programs on disadvantaged children, identified with contextual criteria such as low socio-economic status and free/reduced-price lunches [32,49,50,75,76,78], belonging to ethnic minorities [49,50,76], and living in unsafe and violent neighborhoods [50]. Parental factors including education level [75], mental health issues, and inappropriate parenting practices [76] have also been considered. On the other hand, children with diagnosed mental health problems, special education needs, and disabilities have been scarcely included in the at-risk category [48,75,76], despite it being necessary to pay attention to them to minimize negative consequences, especially in the first years of life [1].

In light of these premises, further research is needed to develop and deliver universal programs adopting a whole-school approach that includes activities to both promoting preschoolers’ competencies and reducing their behavioral problems. PROMEHS was intended to fill this gap. The program was developed within the Erasmus+ Key Action 3 project entitled “Promoting Mental Health at Schools” (PROMEHS), co-funded by the European Commission. The aim of the project was to develop, implement, and evaluate a school-based, universal mental health curriculum for European students from 3 to 18 years of age. The project involved researchers, policymakers, and stakeholders from seven countries, namely Italy, Malta, Latvia, Croatia, Greece, Romania, and Portugal. The effectiveness of the PROMEHS program has already been assessed in relation to the entire international sample of teachers [79] and students from kindergarten through high school [80], Greek [81] and Portuguese [82] students from kindergarten through high school, and Romanian highschoolers [83]. The effectiveness of implementing PROMEHS with preschoolers specifically has only been documented with Latvian children [46].

The PROMEHS program offers multiple positive features, which reflect the highest, state-of-the-art standards for evidence-based interventions [84]. For example, as earlier stated, it was designed to both foster competence and reduce mental health issues. Indeed, the theoretical framework within which the program was developed covers three themes: the promotion of social-emotional competencies, the promotion of resilience, and the prevention of social, emotional, and behavioral problems [34]. This framework also envisages a whole-school approach, whereby key stakeholders in children’s mental health (i.e., teachers, school leaders, and parents) are involved in the intervention. More specifically, the program consists of a training course and supervision sessions for teachers to ensure that they assimilate the theoretical concepts informing the program and reliably implement the relative practical activities in the classroom. This means that the teachers are empowered to deliver the program firsthand, with no direct involvement on the part of the researchers. The program also targets school leaders and parents, through dedicated encounters designed to provide them with knowledge and practical strategies for promoting children’s mental health at school and at home. Involving all these parties enables a joint effort whereby all the adults with a significant role in the children’s education and care can adopt current best practices, thus facilitating the children’s acquisition of competencies and adoption of positive behaviors across different settings [49,85,86]. Another strength of PROMEHS is that the children themselves are actively involved, which further increases the program’s chances of being effective [32]. Specifically, the program entails an experiential approach to learning, such that the children are informed by their teachers about the aims of the classroom activities and are engaged in the learning process by means of self-reflection exercises, games, group discussions, and other practical activities, as illustrated in the ad hoc handbooks. The key features of PROMEHS are summarized in Appendix A.

### 1.4. The Present Study

Given the need for high-quality, evidence-based intervention programs for early years education settings [32,51,87], the aim of this study was to evaluate the effectiveness of PROMEHS when implemented with preschoolers in terms of (1) whether the preschoolers that participated in the program would display gains in their SEL competencies and prosocial conduct, and reductions in their internalizing and externalizing behaviors; (2) because the literature suggests that SEL programs can enhance school success, whether PROMEHS would lead to gains in the preschoolers’ academic outcomes; (3) whether dosage (i.e., the number of program activities actually conducted at school) impacted the effectiveness of PROMEHS; and (4) whether the program yielded greater benefits for at-risk (i.e., marginalized and disadvantaged) children, who typically draw greater gains from participating in SEL interventions.

We hypothesized that the preschoolers who participated in PROMEHS would benefit from it in terms of increased SEL, positive conduct (i.e., prosocial behavior), and school success. We expected that the children’s mental health issues (i.e., internalizing and externalizing problems) would diminish following participation in the program. We predicted that these positive effects would vary as a function of the number of program activities delivered in the classroom, such that children who had the opportunity to engage in a higher number of practical activities would draw greater benefit from taking part in the program. Finally, we hypothesized that at-risk children would display greater improvements following participation in the program than would their less-disadvantaged peers.

## 2. Materials and Methods

### 2.1. Participants

The initial sample consisted of 829 participants, but 116 children (14%) dropped out because they moved to another school or teachers did not complete the post-test, so they were excluded from the dataset. Therefore, participants in the study were 784 preschoolers (413 girls) aged between 4 and 5 years. They were recruited at 21 public or private kindergartens in Italy (12 school clusters, 439 children) and Portugal (9 school clusters, 345 children), after a formal agreement had been stipulated with the school principals. School clusters are the most common structures in both Italian and Portuguese education systems. They are multi-site schools, with a headquarter in the most populous town and different school sites in the surrounding smaller towns. They host children from kindergarten to middle secondary school. Schools of a cluster have the same principal and share the same organizational and administrative framework, driven by the guidelines of the Ministry of Education. Given this similar structure of Italian and Portuguese schools, we decided to focus on preschoolers in these two countries in the current study, within a wider schedule of data analyses that involve the whole PROMEHS network.

Participants who belonged to a marginalized or disadvantaged group (e.g., low socio-economic status, ethnic minority, disability, etc.) represented 14.1% (*n* = 110) of the sample, with 10.2% (*n* = 70) of the sample suffering from a degree of marginalization or disadvantage that was rated by their teachers as quite or very severe (e.g., children with a diagnosed disorder, an individualized educational plan, and a special-needs teacher). In addition to the preschoolers, the study also involved 107 teachers (99% women) from across the 21 kindergartens. Most of the teachers (48.9%) were aged 50 years or over, 26% were aged 40–49 years, 19.8% were aged 30–39 years, and only 5.2% ranged between 18 and 29 years of age. In terms of teacher–student contact, the majority of the children (71.6%) spent 9 h or more with the participating teachers; 24% of children spent between 5 and 8 h with their teacher and only 4.4% spent less than 4 h per week. Schools—not the participants—were randomly assigned to the experimental group (11 school clusters; 6 Italian and 5 Portuguese schools) or waiting list control group (10 school clusters; 6 Italian and 4 Portuguese schools), which numbered 519 and 265 children, respectively. Participants received no financial rewards for joining the study and were free to withdraw at any time.

### 2.2. Research Design and Procedure

The study consisted of three phases: pre-test, intervention, and post-test. The pre- and post-testing was conducted at the beginning and end of the 2020/2021 school year, respectively. The intervention consisted of (A) teacher training; (B) implementation of the classroom curriculum; (C) implementation of the home curriculum; (D) teacher supervision; (E) parent training; (F) meetings with headteachers. Each of these components is described in depth below.

(A) The teachers in the experimental group received 16 h of online training over a one/two-month period (2–4 h per week, according to teachers’ school commitments). They were presented with theoretical knowledge about the mental health of teachers and students (i.e., definition and main facts, the impact of the COVID-19 pandemic, stress and burnout, improving teachers’ social-emotional competencies and resilience), provided with a set of PROMEHS manuals, and instructed about the practical aspects of implementing the PROMEHS curriculum (i.e., examples of PROMEHS practical activities to be carried out with children at school and home). The training sessions were interactive and gave the teachers the opportunity to reflect on the training contents both individually and with colleagues. During the last session, teachers rated the usefulness of the training and their readiness to implement the program [88]. The trainers were psychologists, psychomotor therapists, and education specialists with expertise in teacher training and mental health promotion in school settings. They also completed some monitoring measures before, during, and at the end of the teacher training [88].

(B) Next, the teachers implemented the PROMEHS curriculum with their students in the classroom over twelve weeks, aiming to carry out one handbook activity per week. They implemented the activities when all or most children were present in the classroom, so the attendance rate was always high (90–95%). Among the 29 activities of the handbook, teachers were free to choose the ones that best targeted their students’ needs or were in harmony with the current academic content. The activities featured storytelling, games, songs, role-playing, etc., to support children’s SEL (e.g., understanding the relationship between emotions, thoughts, and behaviors; improving effective strategies to manage emotions; developing perspective-taking and empathy; managing disagreements and solving conflicts with others; understanding and respecting norms and rules) and the acquisition of resilience skills (e.g., to deal with challenges, such as negative peer pressure, and traumatic experiences, such as loss and bereavement), and to prevent social, emotional, and behavioral problems (e.g., anxiety and school phobia, hyperactivity, at-risk behaviors that can cause body injuries). Each activity lasted approximately 1–2 h (see an example of PROMEHS activity for the classroom setting in File S1 [89]).

(C) To further strengthen the children’s acquisition of the target competencies, over the twelve-week period teachers also asked the parents to engage their children in the activities outlined in the home handbook (see an example of PROMEHS activity to be carried out at home in File S2 [90]). The home activities were connected to the ones implemented in the classroom and addressed the same skills. All home activities had a similar structure, that is a brief introduction about the activity that was carried out at school followed by the request to engage in parent–child conversations combined with a drawing, game, cartoon movie, worksheet, etc., to transfer the new skills also into extra-school settings. Each activity approximately required 30 min to be completed.

(D) To ensure that the PROMEHS program was implemented as intended, the trainers set up three/four supervision sessions (approximately one per month) with groups of teachers, for a total of 9 h. The purpose of these encounters was to learn what activities the teachers had carried out with children, what activities children had carried out with their parents and then had been discussed in the classroom, what challenges teachers had met, and what changes they observed, and to provide any support the teachers required. Both qualitative and quantitative measures as checklists, self-reflection forms, and questionnaires were adopted during these sessions to monitor teachers’ and trainers’ work [88].

(E) To enhance the program’s effectiveness, we adopted a systemic approach. Thus, in addition to directly involving the children’s teachers, the trainers also dedicated six hours to individual meetings with the headteacher of each of the schools. These meetings occurred on-site at school or remotely through phone calls approximately once per month. Trainers provided headteachers with theoretical knowledge about school mental health (i.e., definition and main facts, the impact of the Covid-19 pandemic, existing educational policies), briefed them about the importance of school mental health and means of supporting it (e.g., improving teachers’ mental health and school climate, reflecting on actions to enable a change process).

(F) At each school, trainers also scheduled three online sessions with parents (one per month, with a total duration of six hours) with a view to legitimizing mental health and engaging families in the implementation of PROMEHS activities. These webinars were interactive and gave the parents the opportunity to reflect on their skills and competencies. The first session aimed to illustrate the school mental health framework and to understand the impact of the Covid-19 pandemic. During the second webinar, parents were asked to reflect on the best practices to support mental health at home (e.g., improving their social-emotional competencies, and being aware of their emotional socialization practices). Finally, the last session pointed to showing how to implement the PROMEHS curriculum at home through examples of practical activities. During this webinar, teachers were also asked to rate the usefulness of meetings and handbooks and to identify changes they observed in their children [88]. The attendance rate of parents at the three sessions was 41% (213 parents).

Overall, the intervention lasted six months from December 2020 to May 2021. To ensure the fidelity and the quality of the program, the same general procedure and training content was deployed in the two countries. Following the monitoring protocol of the PROMEHS program, both teachers and trainers were asked to complete checklists, scales, and other measures (open- and closed-ended questions) to track their adherence to planned activities [88].

### 2.3. Measures

After providing their informed consent, the teachers completed three online questionnaires about children. The details of these instruments are as follows:

*Social Skills Improvement System, Social Emotional Learning Edition Brief Scales—Student Form* (SSIS-SELb-S) [91]. This questionnaire assesses children’s SEL competencies and comprises 20 items rated on a 4-point Likert scale (from 1, “not true” to 4, “very true”). It yields a composite score ranging from 20 to 80, and five sub-scores corresponding to the different domains of SEL, namely Self-Awareness, Self-Management, Social Awareness, Relationship Skills, and Responsible Decision-Making (with the score for each ranging from 4 to 16). In the case of the original instrument, Cronbach’s alpha reliability coefficients were 0.91 for the composite score, and between 0.67 and 0.72 for the five subscales [92,93]. In the current study, at the pre-test stage, we obtained Cronbach’s alphas of 0.94 for the composite score and between 0.74 and 0.83 for the five subscales. At post-test, Cronbach’s alphas were 0.94 for the composite score and between 0.73 and 0.83 for the five subscales.

*Strengths and Difficulties Questionnaire* (SDQ) [94]. This instrument measures children’s mental health and consists of 25 items to be rated on a 3-point Likert scale (from 0, “not true” to 2, “certainly true”). It yields three scores: for Prosocial behavior (5 items; score ranging from 0 to 10), Internalizing problems (10 items; score ranging from 0 to 20), and Externalizing problems (10 items; score ranging from 0 to 20). The Cronbach’s alphas obtained for the original scales were 0.66 for Prosocial behavior, 0.66 for Internalizing problems, and 0.76 for Externalizing problems [95]. At the pre-test stage of the current study, Cronbach’s alphas were 0.90 for Prosocial behavior, 0.93 for Internalizing problems, and 0.92 for Externalizing problems. At post-test, Cronbach’s alphas were 0.92 for Prosocial behavior, 0.94 for Internalizing problems, and 0.93 for Externalizing problems.

*Academic Outcomes Questionnaire*. We developed this scale ad hoc to measure three dimensions of children’s learning outcomes via three items asking the teacher to rate each students’ academic learning in terms of Motivation at school, Engagement with the learning process, and Academic performance, on a 5-point Likert scale (from 1, “very weak” to 5, “very good”) [80]. In addition to the three sub-scores, the scale also yields a composite score (α = 0.95 both at pre- and post-test).

### 2.4. Data Analysis

All statistical analyses were performed using SPSS Version 28 (IBM Corp., Armonk, NY, USA). Children were matched by code to combine the pre- and post-test scores. Prior to analyzing the efficacy of PROMEHS, we conducted standard data-cleaning procedures. The number of missing values was under 3%, thus the listwise deletion approach was adopted [96]. Furthermore, we assessed the distribution of the data for each of the study measures. None of the kurtosis or skewness values exceeded the recommended limits [−1, +1]. Next, we computed the main descriptive statistics and zero-order correlations. In order to verify whether the two groups were equivalent prior to the intervention, we ran a series of analyses of variance (ANOVAs) to compare the children’s performances at pre-test as a function of group condition. To verify the impact of the intervention on children’s SEL competencies, prosocial behavior, problem behaviors, and academic outcomes we ran a series of repeated-measures multivariate analysis of variance (MANCOVAs) with the following independent variables: time (pre-test or post-test) as a within-subject factor and group condition (experimental or waiting list group) as a between-subject factor. The dependent variables measured at two-time points were SEL competencies (total score and five subscale scores), prosocial behavior, internalizing and externalizing problems, and academic outcomes (total score and three subscale scores). Gender was included as a covariate.

We next conducted correlational analyses to investigate whether the improvement displayed by the experimental group following the intervention was related to dosage of the program—in terms of the number of activities effectively implemented with the participating children. We calculated pre- to post-test changes in the variables under study by subtracting children’s pre-test scores from post-test scores.

Finally, a series of ANOVAs was conducted to test the effect of the intervention as a function of participants’ risk status (marginalized and disadvantaged or not).

## 3. Results

Descriptive statistics are reported in Table 1. The experimental and waiting list groups differed significantly at pre-test in terms of self-awareness, *F*(1,778) = 9.22, *p* = 0.002, social awareness, *F*(1,778) = 10.38, *p* = 0.001, and internalizing problems, *F*(1,778) = 7.43, *p* = 0.007. Table 2 reports the correlations among the variables at pre-test, most of which were statistically significant and strong.

Concerning the effect of the intervention on changes in SEL competencies, there was a significant Time × Group interaction, Wilks’ λ = 0.96, *F*(6,724) = 5.02, *p* < 0.001, η_p_^2^ = 0.04, but no significant effects of gender emerged. The univariate tests revealed that the experimental group outperformed the waiting list group from pre- to post-test in terms of their global performance on the SSIS, *F*(1,729) = 15.01, *p* < 0.001, η_p_^2^ = 0.02, and the following four SEL dimensions: self-awareness, *F*(1,729) = 17.27, *p* < 0.001, η_p_^2^ = 0.02, self-management, *F*(1,729) = 12.51, *p* < 0.001, η_p_^2^ = 0.02, social awareness, *F*(1,729) = 8.85, *p* = 0.003, η_p_^2^ = 0.01, and responsible decision-making, *F*(1,729) = 10.46, *p* = 0.001, η_p_^2^ = 0.01.

Regarding the effect of the PROMEHS program on children’s SDQ scores, we found a significant Time x Group interaction, Wilks’ λ = 0.98, *F*(3,722) = 3.36, *p* = 0.02, η_p_^2^ = 0.01. The univariate tests showed that this interaction was significant for prosocial behavior only, *F*(1,724) = 9.04, *p* = 0.003, η_p_^2^ = 0.01, with the children in the experimental group displaying greater pre- to post-test improvement than did the participants in the waiting list group. No significant effects of gender emerged.

Regarding academic outcomes, again there was a Time x Group interaction, Wilks’ λ = 0.96, *F*(4,742) = 6.85, *p* < 0.001, η_p_^2^ = 0.04, with no significant gender effects. The univariate tests indicated that the Time x Group interaction was significant for the global academic outcomes, *F*(1,745) = 7.06, *p* = 0.008, η_p_^2^ = 0.01, and for the dimensions of engagement in learning, *F*(1,745) = 9.76, *p* = 0.002, η_p_^2^ = 0.01, and academic performance, *F*(1,745) = 13.93, *p* < 0.001, η_p_^2^ = 0.02. Again, the children in the experimental group displayed greater pre- to post-test improvement than did their peers in the waiting list group.

Concerning the effects of program dosage, on average teachers delivered 8.1 activities (SD = 4.45; 27.9% of activities from the PROMEHS curriculum). Table 3 shows the correlations between the mean number of PROMEHS activities carried out at a school and the children’s pre- to post-test changes (calculated by subtracting pre-test scores from post-test scores). A higher number of activities was significantly associated with a greater improvement in SEL competencies and a reduction in internalizing and externalizing behaviors. No significant associations were found between the number of activities implemented and improvements in prosocial behavior or academic outcomes.

**Figure 1:**
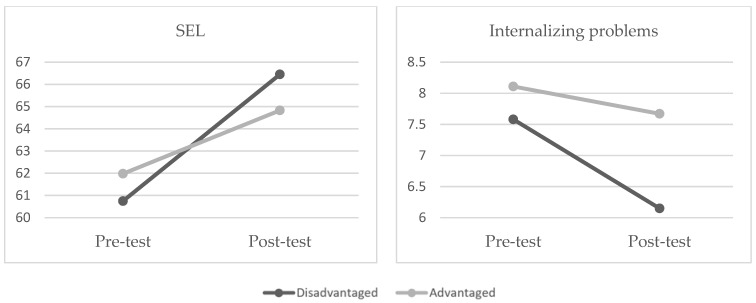
Changes in SEL and internalizing problems in disadvantaged vs. advantaged children from pre- to post-test.

Finally, we compared participants’ risk status within the experimental group. Descriptive statistics are reported in Table 4. Marginalized and disadvantaged children (*n* = 71) displayed significantly greater gains in SEL competencies (global score), *F*(1,466) = 5.07, *p* = 0.025 (M = 5.71, SD = 10.29 vs. M = 2.89, SD = 8.47), and a significantly greater reduction in internalizing problems, *F*(1,474) = 10.07, *p* = 0.002 (M = −1.42, SD = 2.28 vs. M = −0.45, SD = 2.19), than those who were not from a disadvantaged background (Figure 1).

## 4. Discussion

The main goal of this study was to evaluate the effectiveness of the PROMEHS program in a sample of Italian and Portuguese preschoolers by applying a quasi-experimental design. In keeping with our specific research aims, we obtained four main findings that we now discuss in turn. First, as expected, PROMEHS improved children’s SEL competencies and prosocial behavior. School-based SEL programs and interventions have been reported to have positive effects on children, especially when they are implemented with preschoolers [31,32,46]. Large gains in SEL competencies are often documented [50,51], an outcome that we replicated in this study. Indeed, PROMEHS positively impacted almost all the SEL competencies, and namely self-awareness, self-management, social awareness, and responsible decision-making. The children’s active involvement in the program’s practical activities may have provided them with the opportunity to engage in in-depth reflection—as well as in discussion with their teachers and peers—about the attitudes, behaviors, feelings, and thoughts they had experienced, and then to transfer this learning to their everyday lives at school (e.g., when they feel intense emotions or face a problem) and even to other settings (e.g., at home or when socializing with peers outside school). However, the ability to create, maintain, and repair relationships was not affected by the program. This finding may depend on the historical period when PROMEHS was implemented, namely, during the 2020/2021 school year. At that time, strict COVID-19 restrictions were in place in Italian and Portuguese schools; kindergartens were open, but the children were not allowed to play together, interact, or share activities at close range. Thus, it is likely that they did not have many possibilities to daily practice and internalize relationship skills. Further implementation of the program in the present, now that the COVID-19 public health emergency has ended, will disentangle this issue, and show whether or not PROMEHS offers the potential to impact relational competence. As overall effect sizes obtained in the current study were small, ranging between 0.01 and 0.04, the pandemic likely had multiple effects on the program’s outcomes. Based upon previous studies and meta-analyses on universal interventions for preschoolers, we expected moderate effects [32,48], especially because both teachers and parents were directly involved in the program and activities were delivered at school and home [49,50,75]. During the PROMEHS implementation, however, parents’ engagement was limited, and this could have impacted the effectiveness of the intervention.

Furthermore, participation in the program led the children to engage in more prosocial behaviors, even if again the effect size was small (0.01). Teachers reported that their students were more inclined to be kind and respectful of others’ feelings, share toys or materials, and comfort and help other children or adults following the implementation of PROMEHS. This outcome is in line with previous studies that found SEL programs to foster positive behaviors in children [50]. Given that social-emotional competencies are strongly and positively associated with prosocial behavior [36,38,39,40], it is not surprising that both these areas of competencies were impacted by PROMEHS.

On the other hand, unexpectedly, no significant changes were found with respect to internalizing and externalizing problems. However, previous SEL interventions and programs were associated with a small reduction in negative behaviors [49], and proximal effects (e.g., on SEL competencies) have been more frequently reported than distal effects (e.g., on challenging behaviors) [73]. Thus, the findings of the current work are not necessarily inconsistent with past evidence reported in the literature. Indeed, PROMEHS led to decreases in both children’s internalizing and externalizing problems, as borne out by the mean scores of the experimental group at post-test compared to pre-test; on the contrary, emotional and behavioral problems increased slightly in the waiting list group. Therefore, PROMEHS seems to have the potential to prevent children from displaying problematic behaviors. Although these changes were not statistically significant in the present study, this is a pattern that future iterations of the program may confirm.

The second main finding was that PROMEHS, as hypothesized, enhanced children’s academic outcomes. This is in keeping with past findings that SEL interventions can also produce distal outcomes [66]. Indeed, the development of social-emotional competencies is positively associated with pre-academic skills and attitudes in the classroom, such as participation in school activities and motivation [60,62]. Specifically, PROMEHS increased children’s engagement in the learning process and academic performance. It is likely that the social-emotional competencies fostered by the program provided the children with helpful tools and strategies that they were able to use at school. For example, gains in self-management competencies may have enabled the participants to exert greater control over their emotions, behaviors, and so on, thus enhancing their involvement in classroom activities and success in school tasks. On the other hand, the active learning approach that characterized the program may have made the children feel listened to and safe, with positive repercussions on classroom climate and learning [97]. Noteworthy, the effect size was small (from 0.01 to 0.04), which could be explained by the large sample size in the current study that may have led to detect modest effects [66].

Third, we found that the children who took part in a greater number of PROMEHS activities displayed greater gains in SEL competencies and greater reductions in mental health issues (both internalizing and externalizing problems). The teachers were asked to implement the program in the classroom, integrating the activities into the regular curriculum, and to carry out approximately one practical activity per week. However, it was not always possible for them to reach this target during the COVID-19 emergency. Indeed, they faced intermittent school closures due to positive cases among the children or teaching staff and found it challenging to conduct the program activities remotely with their young students. Nevertheless, despite these difficulties, PROMEHS produced positive effects on the children and showed that delivering a greater number of activities can be a protective factor and prevent the onset or worsening of emotional and behavioral problems. As observed by January et al., children who are exposed to interventions for longer and more intensively have greater opportunities to put their new skills into practice and thus, to maintain changes over time [32]. This finding offers support for implementing PROMEHS over a longer period. For example, teachers could deliver program activities for an entire school year. Future applications of PROMEHS will clarify how often the activities should be conducted to maximize positive effects.

Finally, we found that PROMEHS was more effective for marginalized and disadvantaged children than it was for their advantaged peers. Specifically, at-risk preschoolers (i.e., those with low socio-economic status, from an ethnic minority, having special educational needs, etc.) who participated in the program displayed greater gains in SEL competencies and greater reductions in internalizing problems (as rated by their teachers) compared to their non-disadvantaged peers. Although PROMEHS is a school-based universal intervention, which means that it targets all students, it appears to have a greater impact on vulnerable populations, in keeping with previous findings reported in the literature [7]. The program was not effective in reducing emotional and behavioral problems in all children, but it is encouraging that it produced a significant change in a sub-sample of at-risk subjects, who are more prone to internalizing problems than the rest of the school community. It is noteworthy that several recent works have reported that the effectiveness of universal interventions is irrespective of children’s risk status [32,48,49,50,75,76]. However, differently from most previous research, the criteria for considering children as disadvantaged included contextual (e.g., low SES) and individual factors (e.g., special educational needs) in our study. The broadening of “at-risk children” conceptualization may have influenced our findings and points to the need for more research to understand which children are most likely to benefit from school-based universal interventions.

Despite the promising outcomes associated with implementation of the PROMEHS program in this study, some limitations need to be acknowledged. First, the study was carried out during the COVID-19 pandemic, an emergency period characterized by fear of contagion, confinement, and changes in daily routines that impacted the wellbeing of the children and their adult caregivers [98,99,100]. Therefore, this study provided evidence of the effectiveness of the program during a particularly stressful and “atypical” historical period. In relation to this point, we can hypothesize that resilience skills were being deployed and mediated the effects of PROMEHS on children’s competencies and behaviors. Future studies may clarify the role played by children’s resilience and whether the same findings may be obtained in a different historical world context. Follow-up research may also shed light on the persistence of beneficial effects over time.

Second, we relied only on teachers’ ratings of their students, which could have been biased by the fact that they had also delivered the program. The social desirability and the eagerness to see concrete evidence of the effectiveness of their work may have affected their answers. Future research should also include outsiders’ (e.g., researchers not involved in the training) evaluations of children, such as questionnaires, direct testing, or observations, as well as parents’ assessment of their children to check whether they converge with teachers’ responses. Furthermore, children’s improvements in terms of competencies and behaviors may reflect changes in their teachers and parents, due to their direct involvement in the program. There is no doubt that teachers modified their attitudes, as their social-emotional competencies, self-efficacy, and resilience increased after participation in the PROMEHS program [79], with plausible positive effects on children. Nevertheless, changes in parents’ competencies and behaviors due to their participation in the program (i.e., webinars and home activities) have not been assessed. In the current work, parents’ attendance to the webinars was lower than the one reported in other programs [75,76], likely due to the pandemic that prevented higher participation, and we collected only indirect proofs of home activities (i.e., from teachers during the supervision sessions). Future studies should control parental involvement more carefully and adopt specific measures to evaluate parents’ changes, considering their contribution to children’s development and skills transfer especially in the first years of life [2,3].

A third limitation lies in the analyses that we ran. On one hand, we could not investigate age differences because the kindergarten teachers were not asked to provide individual students’ precise ages in the questionnaires. However, this is only a marginal weakness because the children were all between 4 and 5 years old only, which is a limited age range, and they all carried out the same practical activities at school. Indeed, there is only one set of PROMEHS activities for kindergarten (i.e., for children aged 3–5 years). Furthermore, in some of the smaller participating schools, classes were mixed, with younger and older kindergartners participating in the same group. Future research should take this limitation into account and verify whether there are age–related differences in the effects of the program. On the other hand, children’s clustering in schools was not taken into account. Preschoolers’ distribution in the 21 schools was not homogeneous, ranging between 4 and 112 children, resulting in the impossibility to run multilevel analyses. Therefore, further implementation of PROMEHS should account for children’s clustering to clarify which circumstances may make the program more effective. Another issue concerned marginalized and disadvantaged children who participated in the program. This sub-sample was far smaller than the rest of the experimental group (*n* = 71 vs. 424). The low number of these children and the imbalance between the two groups (marginalized/disadvantaged vs. not at-risk) may have affected the results. A larger sub-sample may allow us to reach stronger conclusions in the future.

Despite these drawbacks, the current study offers many strengths. It provides evidence about the effectiveness of a school-based program for children attending kindergarten. As Djamnezhad et al. stated, “there is still a lack of well-designed, high-quality primary studies evaluating SEL-interventions for our youngest children” [87] (p. 9). While the implementation of the program and its assessment were not “perfect”, as our analysis of its limitations makes clear, we did strive to follow current best practice for evidence-based interventions. The preschool years are a valuable period for the development of competencies, abilities, and positive behaviors that will help children to deal with normative and non-normative experiences as they move forward in life. Thus, teachers should be provided with tools for the early implementation of interventions that have been proven effective. PROMEHS is also a universal mental health program with an inclusive principle that targets all preschool children. This kind of intervention is of particular interest because every child can benefit from it in terms of enhanced adjustment, and because it offers a baseline for subsequent targeted interventions for those from deprived social-cultural backgrounds or with specific individual needs [7].

Another strong point of PROMEHS is that its curriculum included specific activities designed to both promote children’s competencies (i.e., SEL competencies and resilience) and prevent problematic behaviors (i.e., internalizing and externalizing problems), whereas the majority of universal interventions are only focused on students’ social-emotional competencies [49]. Last but not least, the PROMEHS program is characterized by a whole-school approach, whereby the entire school community (i.e., children, teachers, parents, and school leaders) is involved. This is of value because all the adults involved in the children’s lives can work collaboratively and in a coordinated way to produce greater effects [49,76,77,85,86].

## 5. Conclusions

The main goal of this study was to evaluate the effectiveness of PROMEHS, a European school mental health program, with Italian and Portuguese preschoolers. Despite the limitations outlined above, the findings were promising, showing that the program can enhance children’s positive outcomes (i.e., SEL competencies, prosocial behavior, and academic learning), especially when a greater number of manualized activities are carried out at school. The effects are also stronger for marginalized and disadvantaged preschoolers, for whom PROMEHS both increases SEL competencies and decreases internalizing problems.

The positive effects associated with implementation of the PROMEHS program suggest the value of early intervention during the preschool years, a critical period for developing key life skills and competencies. As Greenberg recently emphasized in his report on SEL in schools, teachers are often glad to be involved in programs for enhancing their students’ social-emotional competencies, but “they would benefit from improved policies and support from administrators and policymakers to do so effectively” [10] (p. 21). Cost-benefit analyses attest that SEL programs are relatively low-cost and yield significant returns for both school communities and public health economics [101]. Thus, every effort should be made to continue providing evidence on the effectiveness of programs such as PROMEHS (follow-up studies, implementation in new contexts, etc.) so that reliable early intervention can be made available to children on a wide scale.

## Figures and Tables

**Table 1:** Pre- and post-test means and standard deviations for all variables by Group Condition.

	Pre-Test		Post-Test	
	Experimental	Waiting List	Experimental	Waiting List
Self-awareness	11.59 (2.33)	11.06 (2.23)	12.62 (2.14)	11.40 (2.08)
Self-management	11.96 (2.52)	12.25 (2.43)	12.44 (2.39)	12.30 (2.33)
Social awareness	12.42 (2.33)	11.86 (2.31)	13.14 (2.25)	12.13 (2.43)
Relationship skills	12.98 (2.13)	12.81 (2.13)	13.46 (2.05)	13.18 (2.05)
Responsible decision-making	12.85 (2.39)	12.89 (2.72)	13.38 (2.21)	12.98 (2.28)
SEL total	61.78 (10.22)	60.87 (9.54)	65.02 (9.67)	61.99 (9.46)
Prosocial behavior	10.38 (3.13)	10.49 (3.01)	11.06 (3.06)	10.81 (2.99)
Internalizing problems	7.87 (5.09)	8.97 (5.71)	7.48 (5.21)	9.05 (5.23)
Externalizing problems	9.61 (5.23)	9.83 (5.39)	9.31 (5.15)	9.91 (5.26)
Academic motivation	3.90 (0.90)	3.89 (0.88)	4.07 (0.89)	4.04 (0.81)
Engagement in learning	3.87 (0.92)	3.91 (0.88)	4.07 (0.94)	3.94 (0.86)
Academic performance	3.82 (0.93)	3.83 (0.88)	4.06 (0.90)	3.88 (0.87)
Academic outcomes total	11.60 (2.64)	11.63 (2.51)	12.20 (2.63)	11.87 (2.38)

Note. Standard deviations are presented in parentheses.

**Table 2:** Inter-correlations among variables at pre-test.

	1	2	3	4	5	6	7	8	9	10	11	12	13
1. Self-awareness	-												
2. Self-management	0.53 **	-											
3. Social awareness	0.70 **	0.54 **	-										
4. Relationship skills	0.67 **	0.58 **	0.77 **	-									
5. Responsible decision-making	0.70 **	0.73 **	0.72 **	0.79 **	-								
6. SEL total	0.84 **	0.79 **	0.86 **	0.88 **	0.92 **	-							
7. Prosocial behavior	0.31 **	0.42 **	0.35 **	0.34 **	0.45 **	0.44 **	-						
8. Internalizing problems	−0.28 **	0.01	−0.32 **	−0.35 **	−0.15 **	−0.25 **	0.51 **	-					
9. Externalizing problems	−0.39 **	−0.45 **	−0.45 **	−0.45 **	−0.44 **	−0.51 **	0.32 **	0.72 **	-				
10. Academic motivation	0.46 **	0.48 **	0.44 **	0.48 **	0.54 **	0.56 **	0.37 **	−0.12 *	−0.32 **	-			
11. Engagement in learning	0.46 **	0.52 **	0.42 **	0.47 **	0.56 **	0.57 **	0.38 **	−0.08 *	−0.30 **	0.88 **	-		
12. Academic performance	0.45 **	0.50 **	0.38 **	0.46 **	0.55 **	0.55 **	0.38 **	−0.06	−0.28 **	0.84 **	0.88 **	-	
13. Academic outcomes total	0.48 **	0.52 **	0.43 **	0.49 **	0.57 **	0.58 **	0.39 **	−0.09 *	−0.31 **	0.95 **	0.96 **	0.95 **	-
14. Gender	−0.14 **	−0.21 **	−0.16 **	−0.11 *	−0.15 **	−0.18 **	−0.11 *	0.02	0.18 **	−0.22 **	−0.23 **	−0.19 **	−0.23 **

Note. * *p* < 0.01; ** *p* < 0.001.

**Table 3:** Correlation between the average number of PROMEHS activities and pre-to post-test changes in the investigated variables.

	1	2	3	4	5
1. Number of activities	-				
2. SEL changes	0.26 **	-			
3. Prosocial changes	0.02	0.59 **	-		
4. Internalizing changes	−0.20 **	−0.37 **	−0.25 **	-	
5. Externalizing changes	−0.11 *	−0.53 **	−0.39 **	−0.24 **	-
6. Academic outcomes changes	0.07	0.49 **	0.35 **	−0.20 **	−0.37 **

Note. * *p* < 0.01; ** *p* < 0.001.

**Table 4:** Pre- and post-test means and standard deviations for all variables by participants’ risk status.

	Pre-Test		Post-Test	
	Advantaged	Disadvantaged	Advantaged	Disadvantaged
SEL total	61.91 (9.45)	60.75 (13.44)	64.76 (9.39)	66.45 (10.98)
Prosocial behavior	10.77 (3.03)	9.45 (3.52)	11.25 (3.03)	10.44 (3.15)
Internalizing problems	8.29 (5.08)	7.91 (4.71)	7.86 (5.17)	6.35 (4.87)
Externalizing problems	9.96 (5.11)	9.05 (5.76)	9.55 (5.42)	8.27 (5.85)
Academic outcomes total	11.84 (2.57)	10.56 (2.75)	12.36 (2.52)	11.44 (2.83)

Note. Standard deviations are presented in parentheses.

## Data Availability

Research data supporting reported results can be requested from the corresponding author upon reasonable request.

## Supplementary Materials

The following supporting information can be downloaded at: https://www.mdpi.com/article/10.3390/children10061070/s1. File S1: Example of PROMEHS activity—school; File S2: Example of PROMEHS activity—home.

## Appendix A. Main Features of the PROMEHS Program

**Whole-school approach**. PROMEHS acknowledges the importance of collaborative work between students, teachers, families, school leaders, community stakeholders, and policymakers.
**Universal**. The implementation involves the entire school community and does not target only clinical or at-risk populations.
**Multiple initiatives**. The program includes training courses and supervision for teachers; meetings with school leaders, parents, and policymakers; guidelines and manualized activities to be carried out at school and at home; dissemination events for stakeholders.
**Manualized and multi-year handbooks and glossaries**. PROMEHS consists of seven handbooks that offer multi-year programming for students from 3 up to 18 years, their teachers, and parents. Two handbooks include guided activities that students and teachers can carry out at school, as part of the mainstream curriculum (one for kindergarten and primary school, and the other for lower and upper secondary school); two handbooks include guided activities that students can carry out at home, with their parents’ help and contribute. All the activities have been developed according to the S.A.F.E. approach [10]. The other three volumes—addressed to teachers, parents, and school leaders/policymakers—offer guidelines and recommendations for promoting mental health. Furthermore, two glossaries (one for kindergarten and primary school teachers, and one for lower and upper secondary teachers) are provided to support teachers’ mental health literacy. All the materials are available in seven languages (Croatian, English, Greek, Italian, Latvian, Portuguese, and Romanian).
**Professional teachers’ training**. The implementation includes the delivery of high-quality training composed of initial training and ongoing supervision to ensure robust and reliable implementation. Teachers are required to deliver the PROMEHS activities on a weekly basis during the regular school day.
**Themes**. The curriculum comprises three themes: the promotion of SEL; the promotion of resilience; the prevention of social, emotional, and behavioral problems.
**Evidence-based approach**. The program was evaluated by comparing the experimental and waiting list control groups at two-time points and analyzing any significant effects on teachers’ and students’ outcomes. The evaluation was conducted using a sample size that met the ESSA (Every Student Succeeds Act) criterion for offering large enough power to capture the program’s effects [84].
**Independent evaluation**. The independent evaluators, who were not involved in the program’s development and implementation, reviewed the handbooks to reduce potential bias and ensure the reliability of the evaluation procedures and findings.
**Quality of implementation**. The program’s fidelity, dosage, quality, responsiveness, and adaptation are assessed [88].
**Multi-informant assessments**. Multiple informants (students, teachers, and parents) were used to assess the program’s impact on students’ and teachers’ mental health.
**Developmental perspective**. The PROMEHS curriculum acknowledges that students’ and teachers’ mental health encompasses dynamic and multifaceted knowledge, skills, practices, and attitudes that may change over time.
**Active family engagement**. Student and parent handbooks are designed to reinforce the acquisition of skills and behaviors learned at school by applying them at home.
**Sustainability**. The PROMEHS program was developed through collaborative work with local, regional, national, and international policymakers to maximize the impact and sustainability of the results over time.
