# Supplementary material for: Supporting Preschoolers’ Mental Health and Academic Learning through the PROMEHS Program: A Training Study
Source: Children (Basel). 2023 Jun 16;10(6):1070. doi: 10.3390/children10061070 (PMC10297171; doi:10.3390/children10061070)
Supplement: Supplementary file 1 [file children-10-01070-s001.zip › children-2374186-supplementary.pdf]

Goal 1: To be able to identify and label basic and complex emotions

Activity 1: The three little pigs

**Outcome**	At the end of the activity, students will be able to identify, express, and label basic emotions (joy, anger, fear, sadness).
**Targeted group**	Students aged 3 to 5
**Level**	Basic
**Materials**	Crayons, papers, Pc and projector
**Activity steps**	

- The teacher asks students to listen to the story of the "Three Little Pigs". You may use the following video from YouTube: https://www.youtube.com/watch?v=2s7cz6p7jew

Story box

Borna and his friend Lucia decided to watch on YouTube their favourite fairy tale, "Three Little Pigs".
This well-known fairy tale is about three little pigs who decided to build their houses and the difficulties they had during this attempt.

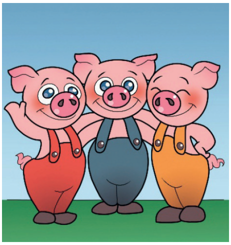

- By the end of the animation, the teacher asks students the following questions:
  1. How do you think the little pigs felt as they were building their homes? What makes you think that?

2. How do you think the first two pigs felt when the wolf destroyed their home? What makes you think that?
3. How do you think the wolf felt when he failed to destroy the last home? What makes you think that?
4. Overall, which emotions did you identify in the animation?

- The teacher divides students in four groups. Each group has to draw and to colour a paper sheet in order to portray one emotion of the following four: joy, anger, fear, sadness. These sheets will be the cards of emotions.
- Then the teacher can mix the cards and assign to each student, in turn, one card following these instruction: those with the green cards must "play" the happy pig, those with the red card "play" the angry bad wolf, those with the blue card "play" the frightened pig, those with the yellow card "play" the sad pig.
- Then, the teacher whispers to a student an emotion (i.e., joy, anger, fear, sadness) and the student must try to show that emotion to the others with a facial expression, a posture, voice or mimics. The student may play the role and the others must find out and tell which emotion he/she is being role played.
- The teacher can use the formative evaluation chart below to observe and assess students' ability to identify and label basic emotions (joy, anger, fear, sadness).

**Formative evaluation chart for teacher**
1. Students are able to identify basic emotions such as joy, anger, fear, sadness in the animation.<br>2. Students are able to label basic emotions such as joy, anger, fear, sadness in the animation.<br>3. Students are able to express basic emotions (joy, anger, fear, sadness) non-verbally (e.g., through posture, voice, mimics).

- The teacher explains the goal of the activity, that is being able to identify, express and label basic emotions (joy, anger, fear, sadness). The teacher can help students identify basic emotions (for themselves and the others) and transfer these skills in different contexts in their life (e.g., to observe and recognise their own emotions as they are playing, as well as their friends emotions).

How can I embed this goal into my teaching subjects?

- You may promote children's ability to identify, express, and label basic emotions during every day routine (e.g., when arriving at school or before leaving, during meals).
- You may promote children's ability to identify, express, and label basic emotions during cooperative play (e.g., when they play with friends) or when they play alone.
- You may promote children's ability to identify, express, and label basic emotions when experiencing success or failure during a motor/physical activity.

Further resources

Books
- *Oops–a-Daisy* (2004)), written by Claire Freedman.
- *The giving tree* (1964), written by Shel Silverstein.
- *Elmer* (1989), written by David McKee.

Movies/Videos
- *Bert – An Adorable Family*. It is a story about a seed seeking its own and meeting rejection from others while doing it, because she is different. https://www.dailymotion.com/video/x2ye63
- *Sesame Street: If You're Happy and You Know It*. It is a song about happiness and how it can be expressed. https://www.youtube.com/watch?v=5015skRvqs8

Goal 1: To be able to identify and label basic emotions

Activity 1: Recognise your emotions

**Outcome**	At the end of the activity, you will be able to recognise, express, and label your basic emotions (joy, anger, fear, sadness).
**Targeted group**	Children aged 3 to 5
**Level**	Basic
**Materials**	Worksheet, pencil or crayon
**Activity steps**	

- Dear parents, at preschool we watched an animation movie and played a game in order to help students identify, express, and label basic emotions (joy, anger, fear, sadness). The animation was based on the well-known story "The Three Little Pigs". You can continue this work by helping your child completing the following activity.
- Encourage your child to answer the following questions:
  1. Do you remember the video "The ThreeL Pigs" you watched at school? What happened in the story?
  2. What emotions did the three little pigs feel?
  3. What emotions (e.g., joy, anger, fear, sadness) did you feel during the movie? Together with your parent, try to express these emotions in front of a mirror.

WORKSHEET "MY EMOTIONS"

When all of us decided to live together, I felt…	◯
When the wolf destroyed my house, I felt …	◯
When the wolf chased me, I felt …	◯
When the wolf failed to destroy my house, I felt …	◯
